# Insurance denials: a peer-to-peer problem in neonatology

**DOI:** 10.1038/s41372-024-01991-7

**Published:** 2024-05-11

**Authors:** Karen D. Fairchild, Scott D. Duncan, Annemarie Stroustrup, Patrick J. McNamara

**Affiliations:** 1 Department of Pediatrics, University of Virginia School of Medicine, Charlottesville, VA USA; 2 Department of Pediatrics, University of Louisville School of Medicine, Louisville, KY USA; 3 Department of Pediatrics, Zucker School of Medicine at Hofstra/Northwell, Hempstead, NY USA; 4 Department of Pediatrics, University of Iowa School of Medicine, Iowa City, IA USA

**Keywords:** Health care economics, Health services

## Background

Increasingly, insurance companies are denying payment for physician-ordered services and therapies [1, 2]. These denials include specific inpatient therapies, specific days of a hospitalization, and denial of coverage for patient transfer and hospitalization for a less acute level of care. The latter creates significant operational dilemmas for NICUs operating at or above capacity. When inpatient care is denied, hospital revenue cycle teams often request a “Peer-to-Peer” conversation (P2P) to allow the ordering physician to explain the medical need for denied services to a physician representative of the insurance company. Academic neonatologists have noted an increase in the number of requested P2Ps and in transfer denials, which encumber an already stressed workforce.

## Methods/Results

As a first step toward gaging the scope and nature of the insurance denial problem in academic NICUs across the United States, a survey regarding P2Ps and transfer denials was disseminated to all 127 members of the Association of Academic Neonatology Division Directors (AANDD). The survey was designed by members of a task force convened by AANDD leadership after an iterative review process and was completed by 60 (47%) AANDD members (19 from NICUs with <50 beds, 32 with 50-100 beds, 8 with >100 beds, and 1 with unit size not indicated). Overall, 58% and 60% of respondents agreed or strongly agreed that P2Ps and transfer denials, respectively, are a significant problem at their institution. Several respondents indicated P2P was not a problem because their hospital administrators decided to “write off” insurance denials rather than requiring attending physicians to spend time on them.

Survey responses are summarized in Table 1. Two predominant themes emerged: (1) the insurance company “peer” is often not a neonatologist, may lack knowledge of neonatology standards, and in some cases the interactions were not considered professional; and (2) the time and effort required to carry out the P2P review detracts from other responsibilities and contributes to physician burn-out. Multiple concerns were associated with denial of transfer to lower acuity care including safety concerns for all patients when the unit is over census, less bed availability to accommodate admission of critically ill patients, and decreased opportunity for breast feeding and parent education leading to delayed NICU discharge. The only survey response that varied according to NICU size was denial of iNO for preterm infants which was noted as a problem by 38% of respondents from mid-size units (50–100 beds) and 58% and 50% of respondents from smaller and larger units, respectively. This finding should be interpreted with caution as it may reflect patient case mix, regional referral patterns, and variability in illness severity in different size units.

**Table 1:** Insurance denial survey results: Association of Academic Neonatology Division Directors.

AANDD Insurance Survey (*n* = 60 respondents)	Response “yes” n (%)
**Strongly agree or agree P2P is a problem**	**35 (58%)**
*Nature of P2P problem*	
Insurance peer is not a neonatologist	41 (68%)
Takes time away from patient care and other duties	46 (77%)
Rarely results in payment for services	14 (23%)
Denial of payment for iNO for preterm infants	27 (45%)
Denial of payment for iNO for infants with diaphragmatic hernia	14 (23%)
**Strongly agree or agree denial of transfer is a problem**	**36 (60%)**
*Consequence of denial of transfer to lower level of care*	
Required to turn away patients when NICU is full	30 (50%)
Breast feeding is more difficult farther from home	29 (48%)
Family visitation is more difficult, increasing family stress	41 (68%)
Family education is more difficult, prolonging length of stay	29 (48%)

*iNO* inhaled nitric oxide.

## Limitations

The analysis was limited by the relatively small number of very large units (more than 100 beds) represented, and by inability to assess the geographic region of survey respondents.

## Conclusions

The increase in insurance denials is creating an operational problem. Although insurance company algorithms are designed to reduce unnecessary and costly care, they often ignore documented benefits of specific therapies or care practices for individual patients. Interestingly, only a minority of respondents indicated that the initial denial is upheld after a P2P, perhaps leading to the notion that these conversations are a waste of physician time (i.e., services should have been covered without a P2P).

We undertook this as a preliminary survey and there is a need for a more granular appraisal of the financial impact and operational burden to the medical workforce. Strategies to mitigate the problem were suggested. *First*, documentation in the medical record of rationale for and response to specific therapies or care plans may allow insurance representatives to accept charges without a P2P [3, 4]. Second, education for insurance representatives in the form of up-to-date publications may lead to a shift in denial of therapies such as iNO that may benefit subpopulations of neonates [4–6]. Third, appeals to insurance companies to pay for transfer of non-acute patients to another hospital for “convalescent care” would open admission beds for higher acuity patients. Finally, national provider organizations should partner with payers to develop standards to ensure consistency and equity of care delivery and remuneration to reduce administrative burden and improve patient outcomes.
