# Correction: Insurance denials: a peer-to-peer problem in neonatology

**DOI:** 10.1038/s41372-024-02028-9

**Published:** 2024-06-12

**Authors:** Karen D. Fairchild, Scott D. Duncan, Annemarie Stroustrup, Patrick J. McNamara

**Affiliations:** 1 Department of Pediatrics, University of Virginia School of Medicine, Charlottesville, VA USA; 2 Department of Pediatrics, University of Louisville School of Medicine, Louisville, KY USA; 3 Department of Pediatrics, Zucker School of Medicine at Hofstra/Northwell, Hempstead, NY USA; 4 Department of Pediatrics, University of Iowa School of Medicine, Iowa City, IA USA

**Keywords:** Health care economics, Health services

Correction to: *Journal of Perinatology* 10.1038/s41372-024-01991-7, published online 11 May 2024

In this article the author’s name Annemarie Stroustrup was incorrectly written as Annemarie Stroustrop.

The original article has been corrected.

